# Serum Golgi Protein 73 (GP73) is a Diagnostic and Prognostic Marker of Chronic HBV Liver Disease

**DOI:** 10.1097/MD.0000000000000659

**Published:** 2015-03-27

**Authors:** Zhengju Xu, Liguan Liu, Xingnan Pan, Kaipeng Wei, Meijuan Wei, Lifei Liu, Huanwen Yang, Qian Liu

**Affiliations:** From the Clinical Liver Center (ZX, Liguan Liu, XP, HY, QL); Central Laboratory of Clinical Hepatology (KW, MW); and Department of Pathology (Lifei Liu), The 180th Hospital of the People's Liberation Army, Quanzhou, China.

## Abstract

Alanine aminotransferase (ALT) is the most commonly used marker of liver injury, but normal ALT levels are seen in a proportion of chronic hepatitis B virus (HBV)-infected patients with severe liver injury. Golgi protein 73 (GP73) is a promising alternative marker of liver injury. This study assessed the relation between GP73 levels and liver disease severity, monitored the kinetic changes in GP73 levels in chronic HBV patients receiving entecavir (ETV) therapy, and investigated the potential diagnostic and prognostic values of serum GP73 as a new liver injury biomarker in chronic HBV infections.

This study enrolled 1150 patients with chronic HBV infections, 200 of whom were retrospectively enrolled in this study after receiving 1 year of ETV treatment. GP73 expression in liver tissue was detected by immunohistochemistry. GP73 levels in single or serial serum samples were measured by enzyme-linked immunosorbent assay.

Immunohistochemical analysis indicated that GP73 protein expression in the liver increased progressively with pathologic progression from nonexistent or mild hepatitis to severe hepatitis and cirrhosis during chronic HBV infection. Serum GP73 levels were positively correlated with the disease severity of chronic HBV infections (*r* = 0.58, *P* < 0.001). In patients with normal ALT levels, serum GP73 concentrations were significantly higher in patients with prominent hepatic inflammatory injury and fibrosis than in patients without hepatic inflammatory injury or fibrosis. Serum GP73 concentrations and GP73 protein expression were decreased in the liver tissues of patients whose ALT levels normalized after 1 year of ETV antiviral therapy.

Changes in serum GP73 levels were closely associated with changes in liver injury severity, and, therefore, GP73 may be an effective new liver inflammatory injury biomarker, and could be useful for monitoring the prognosis of chronic HBV infectious patients with normal ALT levels.

## INTRODUCTION

Chronic hepatitis B virus (HBV) infection represents a major challenge to global public health.^1,2^ More than 240 million people worldwide are chronically infected with HBV, and more than 780,000 people are estimated to die of chronic HBV infection every year.^3^ Chronic HBV infection can trigger hepatic necrosis, inflammation, and fibrosis and can lead to advanced liver diseases, including cirrhosis and hepatocellular carcinoma (HCC).^4,5^ Antiviral therapy is the recommended treatment for chronic HBV infection. Because antiviral treatment rarely cures HBV infection, the primary goals of antiviral therapy are as follows: first, the suppression of HBV replication; second, the prevention of liver disease progression to cirrhosis, decompensated cirrhosis, or HCC; and third, the delay of end-stage liver disease.^4,6^ Alanine aminotransferase (ALT) blood concentration is an important index used to evaluate liver injury and decide if chronic HBV patients should receive antiviral therapy. Unfortunately, the specificity and sensitivity of ALT is insufficient to diagnose all liver injury.^7,8^ For instance, 20% to 30% of chronically infected HBV patients with normal ALT scores have severe necrosis accompanied by prominent inflammation, and some patients have already developed fibrosis.^8^ Therefore, patients with ongoing liver injury who would benefit from antiviral treatment may not be identified because of normal ALT levels. The current “gold standard” for liver pathology detection is liver biopsy.^9^ Biopsy-based pathology detection can guide antiviral treatment decisions,^10^ but the clinical use of this diagnostic technique is limited by the invasiveness and associated risks of the procedure and the difficulty in obtaining patient consent. Therefore, there is an urgent need to identify new biomarkers with improved specificity and sensitivity that can detect liver injury and predict chronic liver disease progression.

Golgi protein 73 (GP73) is a type II Golgi transmembrane protein with an estimated molecular weight of 73 kDa. GP73 is expressed primarily by the bile duct epithelial cells, and rarely by the hepatocytes of the normal human liver.^11,12^ Upregulated GP73 expression was recently reported in HCC patients,^13,14^ and its expression was suggested as a potential HCC serum marker.^15,16^ Subsequent studies reported that hepatic GP73 expression was increased in acute and chronic liver disease,^17^ and serum GP73 concentration elevation was found to be related to chronic liver disease progression,^18,19^ liver inflammation, and fibrosis.^20^ In 1 example study, GP73 expression was significantly upregulated in injured livers, and increased GP73 hepatocyte expression seemed to be an important feature of liver disease progression.^11^ Therefore, evidence indicates that, in addition to being a potential HCC serum marker, serum GP73 is also a potentially useful marker of general liver disease progression.^21^ However, the role of GP73 in chronic HBV infection has not yet been fully investigated. Very few studies have been conducted focused on the dynamic changes in GP73 liver expression and serum levels in chronic hepatitis B (CHB) patients after the initiation of entecavir (ETV) therapy. Chronic HBV infection can cause a wide spectrum of pathologic liver changes from nonexistent to mild through severe hepatitis, to extensive hepatocyte death. We hypothesized that changes in GP73 levels would be associated with changes in the severity of chronic HBV infection associated liver injury. To analyze GP73 expression in liver tissues with a spectrum of liver injuries, we detected GP73 levels in single or serial serum samples from the 1150 chronic HBV infection patients enrolled in this study. In the enrolled patients, we correlated GP73 concentration in the liver and serum with liver injury severity and disease progression. Our results indicate that GP73 may be a useful marker for liver injury and disease progression diagnosis. GP73 could be used as a supplementary marker to improve diagnosis in cases in whom the specificity and sensitivity of ALT are insufficient to establish a definitive diagnosis and to minimize the need for liver biopsy.

## MATERIALS AND METHODS

### Subjects

All study participants were recruited from patients admitted to our clinical liver center (The 180th Hospital of People's Liberation Army, Quanzhou, China) consecutively during the period from January 2012 to October 2014. This prospective study enrolled 1150 patients with chronic HBV infection, including 100 chronic HBV carriers (HBV-C), 550 CHB patients, 250 cases with liver cirrhosis (LC, 200 with decompensated LC), and 250 cases with HBV-related HCC. Fifty healthy individuals were selected to serve as the normal control group during the same period. All patients, but the normal control subjects, were positive for HBV surface antigen (HBsAg) and HBV DNA. Demographic data for all participants are reported in Table 1. This study was approved by the Ethical Review Committee of the 180th Hospital of People's Liberation Army. All participants provided written informed consent.

**TABLE 1:**
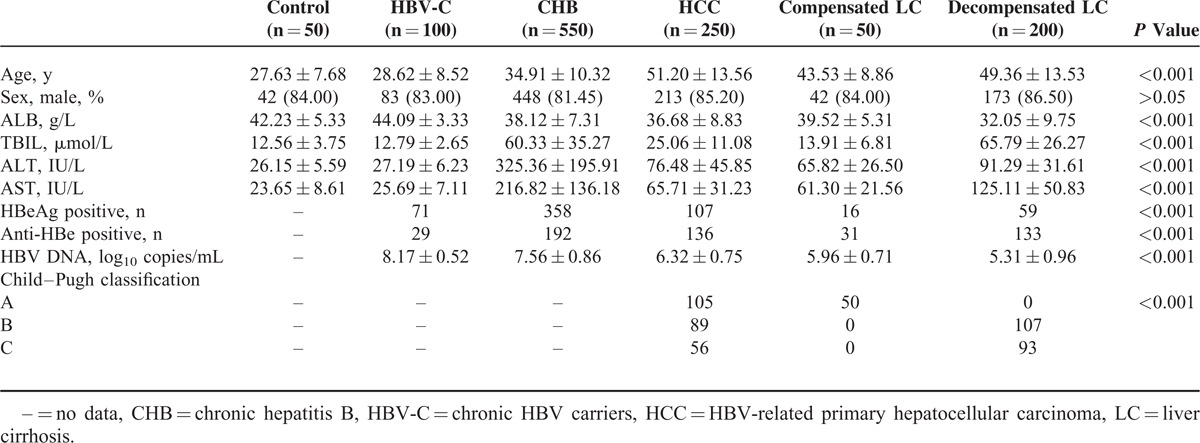
Demographic and Clinical Characteristics of All Participants

Chronic hepatitis B infection diagnosis and categorization was performed according to the guidelines for the prevention and treatment of CHB recommended by the Chinese Association for the Study of Liver Diseases.^22^ HBV-C diagnosis criteria included the following: patients tested HBsAg and HBV DNA positive for more than 6 months during regular clinical follow-up evaluations, and patients had persistently normal ALT (<40 IU/L) and aspartate aminotransferase (AST) levels (<40 IU/L) monitored once every 3 to 6 months for at least 1 year. Patients were categorized as CHB if: they had chronic hepatitis B history or tested HBsAg positive for more than 6 months, and they remained HBsAg and HBV DNA positive at enrollment with persistently or intermittently elevated ALT levels or histologic evidence of hepatic necroinflammation. All patients who fulfilled the clinical criteria were diagnosed with liver cirrhosis and HCC, as established by the American Association for the Study of Liver Diseases.^23^ All patients with HBV-C, CHB, and LC had HCC excluded by ultrasound and alpha-fetoprotein (AFP) examination. HCC diagnosis was confirmed by at least 2 imaging modalities (computed tomography, magnetic resonance imaging, and ultrasound) and AFP ≥ 400 ng/mL. Patients meeting 1 or more of the following exclusion criteria were eliminated from the cohort: first, pregnancy, diabetes, thyroid disease, AIDS, tuberculosis, autoimmune liver diseases and serious cardiac, lung, kidney, nerve, and mental diseases; second, younger than 18 years of age; third, antiviral therapy administered before the screening; fourth, patients with concomitant drug or alcohol-related chronic liver disease, or hepatitis A, C, D, and E; and fifth, HCC patients without chronic HBV infections were excluded from the HCC group. Blood samples were collected from all of the subjects at the time of enrollment. Liver biopsy was performed in 100 patients diagnosed as having HBV-C. Of the 550 patients diagnosed as having CHB, 200 were followed for over 12 months. Blood samples were collected from these 200 patients at enrollment and 1, 3, 6, 9, and 12 months after treatment was initiated. All samples were stored at −80°C for subsequent investigations. Thirty of 200 CHB patients had the liver biopsied twice, one at pretreatment and the other in 1-year post-ETV-treatment. Immunohistochemical (IHC) staining of liver GP73 expression was performed.

In this study, all samples were coded and blind to the readers of the index tests and pathological grading and staging.

### Antiviral Therapy

Patients with CHB received 0.5-mg ETV (Zhengda Tianqing Pharmaceutical Group Ltd., Jiangsu, China) every morning before eating or drinking. Drug withdrawal standards followed EASL clinical practice guidelines.^4^

### Determination of Serum GP73 Level

Quantitative determination of GP73 concentration in serum was performed using a commercially available enzyme-linked immunosorbent assay (ELISA) kit according to the manufacturer's instructions (Beijing Hotgen Biotech Co. Ltd., Beijing, China). Briefly, 20 μL of serum and 50 μL of dilution solution were added to each well of the ELISA microplate, and the microplate was sealed and incubated at 37°C for 60 min. Then, 100 μL of the conjugated antibody to GP73 was added to each well, and the microplate was sealed and incubated at 37°C for 30 min. Next, all solutions were removed and each well was washed 5 times with phosphate-buffered saline/Tween. After washing, 50 μL of the chromogenic substrates A and B was added to each well and the plate was incubated in darkness at 37°C for 15 min. After incubation, 50-μL stop solution was added to each well, and the plate was read using a Bio-Rad 860 microplate reader (Bio-Rad, Berkeley, California, USA). The OD values were converted to ng/mL using the standard curve generated for each plate. Determination of serum GP73 level was performed by the same experienced laboratory technologist.

The coefficient of variation of ranging was from 1% to 15%. The sensitivity (detection limit) of serum GP73 concentration was ≤25 ng/mL. An overall intra-assay coefficient of serum GP73 was ≤15%. The reference interval calculated on 50 healthy subjects by the nonparametric percentile method (CLSI C28-A3) of serum GP73 concentration was 45 ng/mL.

### Liver Pathology

Liver biopsies were obtained using 16G disposable needles (C. R. Bard, Inc., Murray Hill, New Jersey, USA). Liver biopsy specimens were considered reliable when the liver specimen length was ≥1.5 cm or the portal tract number was ≥6 on the section. Specimens were fixed in 4% formaldehyde (Shanghai Xi Hua Trade Co. Ltd., Shanghai, China), embedded in paraffin (Maoming Huayue Group Co. Ltd., Maoming, China), cut into 4-μm sections, and stained with hematoxylin and eosin (Shanghai Xi Hua Trade Co. Ltd.), reticular fiber stain, or Masson trichrome stain (Beijing Sequoia Jinqiao Biological Technology Co. Ltd., Beijing, China). Images were acquired with an Olympus BX51 microscope (Olympus, Tokyo, Japan). The Scheuer scoring system was used to evaluate hepatic necroinflammatory activity and the hepatic fibrosis stage.^24^ The hepatic necroinflammatory grade (G) was divided into grades G0 through G4, and the hepatic fibrosis stage (S) was divided into stages S0 through S4. All liver samples were graded and staged by the same experienced liver pathologist.

### Immunohistochemistry

IHC staining of liver sections was performed using an EliVision Plus IHC kit (Fuzhou Maixin Biotechnology, Fuzhou, China) according to the manufacturer's instructions. Briefly, 4-μm sections were deparaffinized with xylene, and rehydrated through a series of media with descending alcohol concentrations. Antigen retrieval was performed using a high-temperature and high-pressure antigen retrieval method. After rinsing in Tris-buffered saline (pH 7.6), the sections were immersed in 3% H_2_O_2_ to block endogenous peroxidase activity. Samples were then incubated with GP73 monoclonal mouse antibody (Beijing Hotgen Biotech Co., Ltd.) overnight at 4°C. After a Tris-buffered saline wash, HRP-labeling goat anti-mouse (Fuzhou Maixin Biotechnology) was added to each section for 30 min at 37°C, and then visualized using DAB (Beijing Sequoia Jinqiao Biological Technology Co. Ltd.). Counterstaining was performed using hematoxylin. Phosphate-buffered saline without the primary antibody was used as a blank control.

### Evaluation of the IHC Staining

Semi-quantitation of GP73 expression in liver tissues was conducted with a 4-tiered system based on the staining intensity of GP73:^12^ 0 (negative), 1+ (weak), 2++ (moderate), and 3+++ (strong). Weak immunoreactivity was defined as minute brown granules projecting to the Golgi apparatus. Moderate immunoreactivity was diagnosed when a coarser and more intense brown staining was seen. Chunky, dark lumps were scored as strongly positive. IHC staining was reviewed and quantified by the same experienced liver pathologist.

### Biochemical Indexes of Liver Function Tests

The liver function-related biochemical indexes of albumin (ALB), total bilirubin (TBIL), ALT, and AST were determined using the chemical colorimetric method in a TBA-120 FR fully automatic biochemical analyzer (Toshiba, Tokyo) according to the manufacturer's instructions (Beijing Condor-Teco Medical Technology Co., Ltd.; Beijing).

### Statistical Analysis

Statistical analyses were performed with GraphPad Prism version 5.0 (Graphpad Software Inc., La Jolla, California) and SPSS 19.0 software (International Business Machines Corporation, New York, USA). Measured data were expressed as mean ± standard deviation. For means of the data that satisfied the homogeneity of variance, differences were tested with analysis of variance (ANOVA), and data that did not satisfy the homogeneity of variance were analyzed with ANOVA following rank transformation. The serum GP73 values post-ETV-treatment were reanalyzed with ANOVA-repeated measures. Rates of classification data were compared by χ^2^ square test. Correlation analyses among variables were performed using Pearson correlation coefficient (*r*) analysis and linear regression analysis. The correlation between serum GP73 level and the extent of pathologic changes were performed with multivariate logistic analysis. *P*-value < 0.05 was considered statistically significant. The diagnostic performance of GP73 was evaluated by performing an area under the receiver-operating characteristic (ROC) curve analysis with a 95% confidence interval (CI).

## RESULTS

### Baseline Demographic and Clinical Data

From January 2012 to October 2014, 1150 patients with chronic HBV infections were enrolled in this study. Demographic and clinical characteristics at the time of enrollment are shown in Table 1. No significant differences in sex ratios were observed among groups (*P* > 0.05) although significant differences in age, ALB, TBIL, ALT, AST, hepatitis B virus e antigen, hepatitis B virus e antibody, and HBV DNA levels were found among the 5 groups (*P* < 0.001). All but 3 cases had a complete set of blood samples available for serum GP73 follow-up among 200 patients who received ETV treatment for 1 year or longer. There were no indeterminate results, missing data, or outliers of the index tests in all assays.

### Changes in GP73 Liver Tissue Expression

**FIGURE 1:**
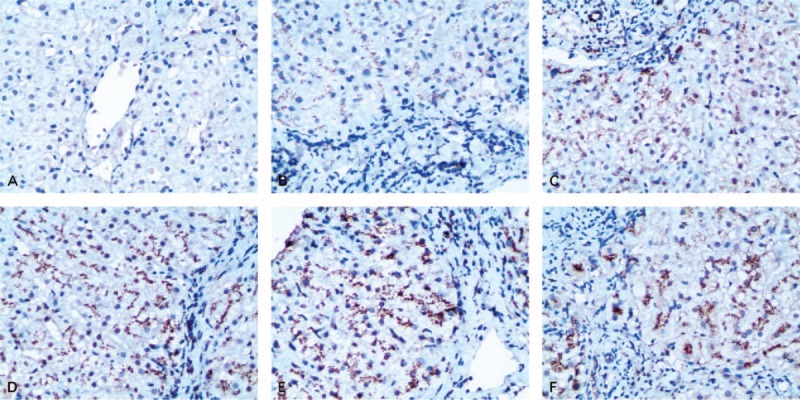
GP73 protein expression in normal and chronic HBV-infected liver tissues. (A) IHC showed that a majority of hepatocytes in the normal liver tissue were negative for GP73 protein expression. (B) Weak GP73 staining was detected in the liver of a HBV-C patient without prominent necroinflammation. (C) Many hepatocytes were detected with moderate GP73 staining in the moderately inflamed liver. Strong GP73 positivity was detected in the liver with severe chronic hepatitis B (D), compensated liver cirrhosis (E), and HCC (F). (Magnification, ×200). HBV-C = HBV carriers, HCC = HBV-related primary hepatocellular carcinoma, IHC = immunohistochemical.

GP73 expression was detected in liver tissues of 16 patients with chronic hepatitis B. IHC staining results showed that a majority of hepatocytes in the normal liver tissue were negative for GP73 protein expression (Figure 1A). In patients with chronic HBV infections, GP73 was expressed with a scattered pattern in the cytoplasm of hepatocytes, but not in the infiltrating inflammatory cells. The detected GP73 positive cell percentages in HBV-C, CHB, severe hepatitis, compensated LC and HCC patients were about 15.00%, 45.00%, 75.00%, and 70.00%, respectively. GP73 expression was generally weak in HBV-C (Figure 1B), moderately positive in CHB (Figure 1C), strongly positive in severe hepatitis (Figure 1D), compensated LC (Figure 1E), and HCC (Figure 1F). Our IHC analysis indicated that GP73 expression and expression intensity in hepatocytes gradually increased with the progression of chronic hepatitis B.

### Serum GP73 Was Elevated in Parallel With Chronic Hepatitis B Progression

**FIGURE 2:**
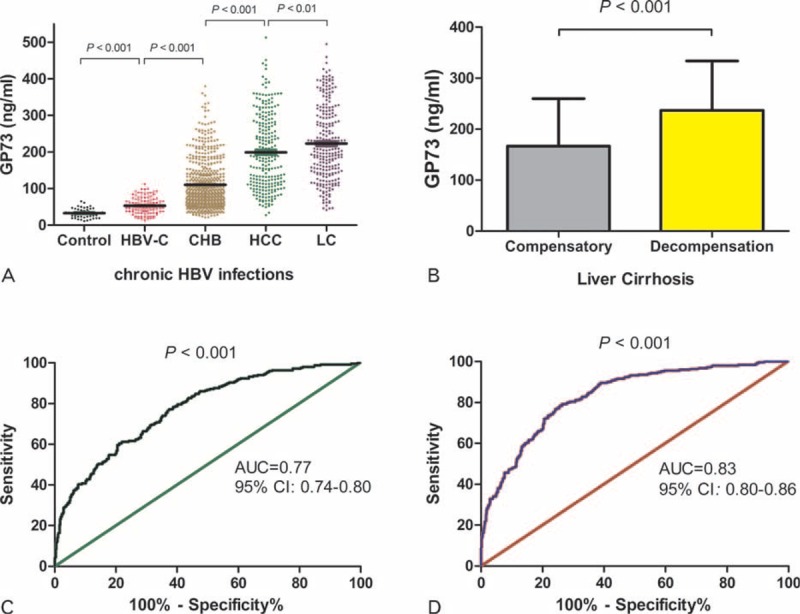
Distribution of quantitatively detected serum GP73 levels in patients with different severities of HBV-related liver diseases. (A) The concentrations of serum GP73 in 5 groups: control = healthy control; HBV-C = HBV carriers; CHB = chronic hepatitis B; HCC = HBV-related primary hepatocellular carcinoma; LC = hepatitis B liver cirrhosis. (B) Serum GP73 levels in decompensated and compensatory LC. (C) ROC curve for the diagnosis of HCC. (D) ROC curve for the diagnosis of LC. AUC = area under ROC curve, CI = confidence interval, ROC = receiver-operating characteristic.

We assessed whether there were serum GP73 changes that were associated with liver disease progression from nonexistent or mild to severe hepatitis, and, furthermore, whether serum GP73 changes were associated with chronic hepatitis B complications. We observed that serum GP73 was significantly increased in chronic HBV infection patients when compared with the healthy control group (*F* = 191.60, *P* < 0.001). Furthermore, along with increasing necroinflammatory and fibrotic severity, serum GP73 increased with the progression of chronic hepatitis B. Serum GP73 levels were 53.15 ± 22.79 ng/mL in HBV-C, 110.19 ± 66.91 ng/mL in CHB, 195.01 ± 104.22 ng/mL in HCC, and 225.71 ± 99.37 ng/mL in LC. Serum GP73 levels were significantly different between all groups (all *P* < 0.001, Figure 2A). Pearson correlation analysis showed that there was a positive correlation between changes in the serum GP73 level and liver disease severity in CHB patients (*r* = 0.58, *P* < 0.001). Serum GP73 levels in patients with decompensated cirrhosis were much higher than in patients with compensatory cirrhosis (236.78 ± 97.15 ng/mL vs. 166.075 ± 93.38 ng/mL) (*P* < 0.001, Figure 2B).

### Sensitivity and Specificity of GP73 for HCC or LC Diagnosis

ROC curve analyses showed that the sensitivity and specificity of GP73 for HCC diagnosis were 72.50% and 68.11%, respectively, in which the cut-off value was set at 153.60 ng/mL. The area under the ROC curve was 0.77 (95% CI: 0.74–0.80) (Figure 2C). If the diagnostic cut-off value was set to 138.20 ng/mL, the sensitivity and specificity of GP73 for LC diagnosis were 79.20% and 80.00%, respectively. The area under the ROC curve was 0.83 (95% CI: 0.80–0.86) (Figure 2D). In the LC subgroup, the sensitivity and specificity of GP73 for predicting decompensated LC were 66.50% and 66.00%, respectively, in which the cut-off value was set at 186.20 ng/mL. The area under the ROC curve was 0.71 (95% CI: 0.62–0.79).

### Dynamic Changes in GP73 Levels Indicate Potential Prognostic Value for CHB Patients

**FIGURE 3:**
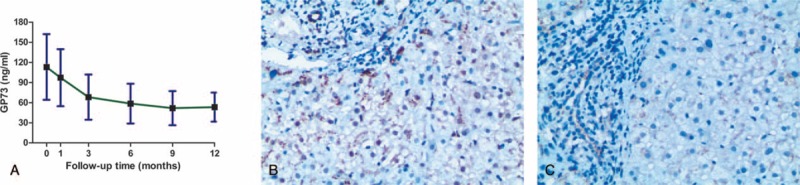
Changes in serum GP73 concentration and intracellular GP73 protein expression level before and after 1 year of ETV therapy. (A) Changes in serum GP73 concentration during ETV antiviral therapy in patients with CHB. (B) Moderately positive GP73 hepatocytes detected before treatment. (C) Weakly positive GP73 detected after 1 year of ETV treatment. (Magnification, ×200). CHB = chronic hepatitis B, ETV = entecavir.

In 200 CHB patients who received ETV treatment for more than 1 year, serum HBV DNA was decreased and biochemical indexes were returned to normal after 3 months. We monitored the dynamic changes in serum GP73 concentration at the start of treatment and 1, 3, 6, 9 and 12 months after initiating the treatment. Along with reduced liver necroinflammation, the serum GP73 concentration also gradually declined from 97.26 ± 42.52 ng/mL in the first month of treatment to 68.21 ± 33.65 ng/mL at month 3, 58.57 ± 29.52 ng/mL at month 6, 51.76 ± 25.39 ng/mL at month 9, and 53.37 ± 21.62 ng/mL at month 12. The average GP73 serum concentration fell significantly from its baseline level (113.09 ± 48.91 ng/mL) compared with its serum concentration after 12 months of treatment (*P* < 0.01). The largest serum GP73 concentration decrease occurred after 3 months of treatment, coinciding with ALT normalization (Figure 3A). GP73 levels continued to decline until stabilizing after month 9 (Figure 3A). Our ANOVA-repeated measures showed that the serum GP73 concentration linearly declined to the normal level during 1-year ETV treatment (linear F = 256.15, *P* < 0.001).

We further compared the changes in liver tissue GP73 expression in 30 cases between pretreatment and 1-year post-ETV-treatment patients. GP73 expression in the liver was clearly reduced by treatment from moderate or strong expression to weak expression (Figure 3B and C). Semiquantitation of GP73 expression showed that the expression difference was significant (*x*^*2*^ = 6.70, *P* < 0.05, Table 2).

**TABLE 2:**
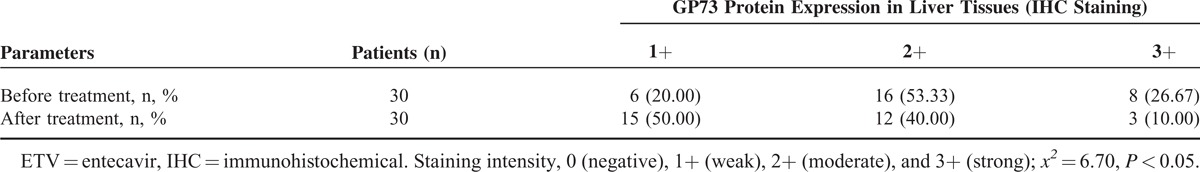
Changes in Hepatic GP73 Expression Before and After 12 mo of ETV Therapy

### Elevated Serum GP73 Was Associated With Increased Pathologic Changes in HBV-C Patient's Livers

**FIGURE 4:**
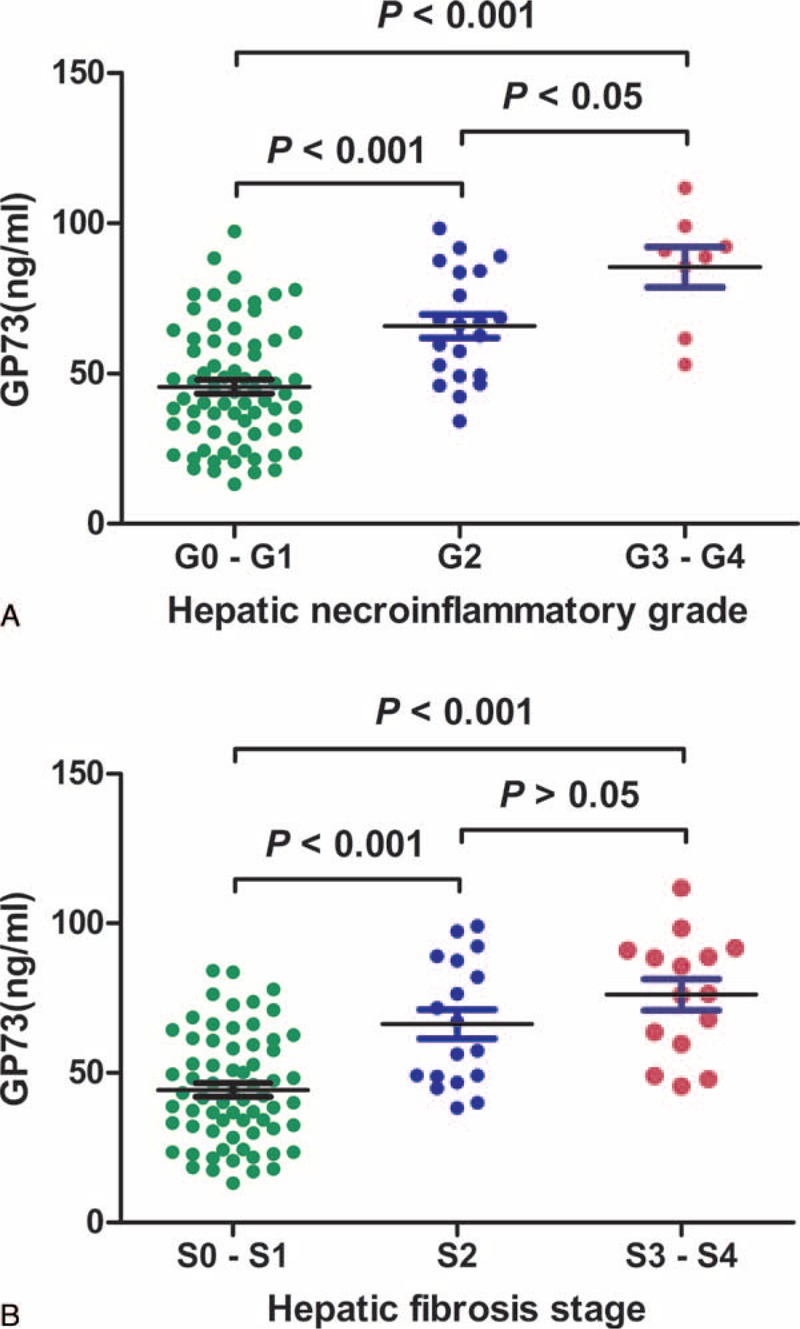
Serum GP73 level correlated with hepatic necroinflammatory grade (G) and hepatic fibrosis stage (S) in patients with HBV-C. HBV-C = HBV carriers.

One-hundred HBV-C patients underwent liver biopsy. Liver pathology scores are shown in Figure 4. We examined the correlation between serum GP73 levels and the extent of pathologic changes, and found that, with the exception of the S3–S4 group, mean serum GP73 concentrations increased significantly with increasing hepatic necroinflammatory grades (G0–G4) and hepatic fibrosis stages (S0–S4) (Figure 4A and B). Serum GP73 levels positively correlated with hepatic necroinflammatory grades (*r* = 0.55, *P* < 0.001) and hepatic fibrosis stages (*r* = 0.53, *P* < 0.001). Out of 100 cases diagnosed as HBV-C with normal ALT, 29 (29%) and 33 (33%) showed prominent necroinflammation (≥G2) and fibrosis (≥S2), respectively. Serum GP73 levels in patients with pathologic grades ≥G2 and ≥S2 were significantly higher than the serum levels of patients with (G0–G1, or no visible necroinflammation) and (S0–S1, or no visible fibrosis) (all *P* < 0.001, Table 3). Serum GP73, ALB, TBIL, and ALT levels in 100 HBV-C cases with normal ALT were subjected to multivariate regression analysis. Only serum GP73 was identified as an independent factor for predicting hepatic inflammation (Wald = 16.33, *P* < 0.001) and fibrosis (Wald = 20.57, *P* < 0.001). Our data suggest that serum GP73 was a more sensitive liver injury biomarker than ALT, and that its expression also reflected the occurrence of hepatic fibrosis in the patients with HBV-C.

**TABLE 3:**
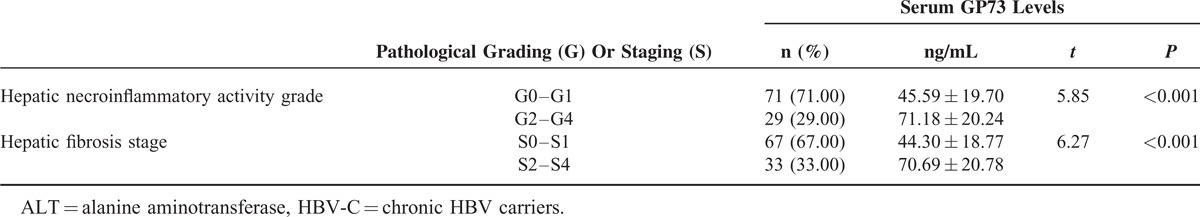
Serum GP73 Level Correlations With Pathological Grading and Staging in HBV-C Patients With Normal ALT Levels

### Associations Between Serum GP73 Levels and Biochemical Indexes

**FIGURE 5:**
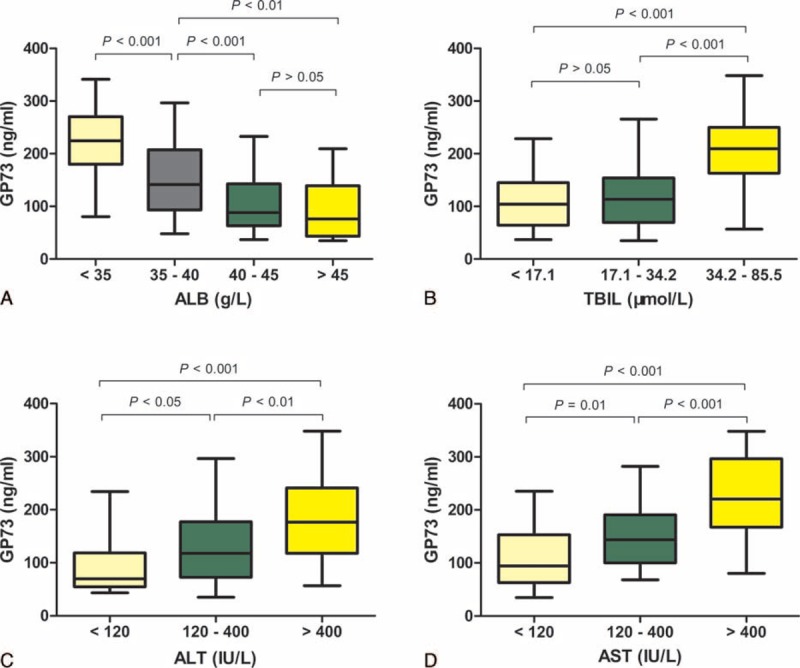
Serum GP73 levels correlated with other biochemical markers. Serum GP73 levels in patients with different ALB (A), TBIL (B), ALT (C), and AST (D) levels.

Associations between serum GP73 levels and the biochemical indexes of ALB, TBIL, ALT, and AST in 550 CHB cases are shown in Figure 5. Serum GP73 levels were negatively correlated with ALB levels (*r* = −0.56, *P* < 0.001), but positively correlated with TBIL levels (*r* = 0.40, *P* < 0.001), ALT levels (*r* = 0.48, *P* = 0.001), and AST levels (*r* = 0.51, *P* < 0.001).

## DISCUSSION

Serum markers are a critically important tool for detecting and predicting liver injury, inflammation, fibrosis, and carcinogenesis in chronic HBV patients. Serum markers are particularly valuable for helping to determine whether antiviral treatment is recommended and for monitoring the antiviral response of patients receiving treatment. Current biomarkers include ALT, ALB, TBIL, and prothrombin time. When evaluating HBV infection patients, these biomarkers, as well as liver fibrosis serum markers, can indicate the extent of liver injury, degree of inflammation, and performance capacity of important liver functions. However, the current biochemical markers for liver injury are not sufficient to meet clinical needs. For instance, a portion of chronic HBV-infected patients, who are categorized as HBV-C by the current diagnostic methods, may have actually experienced serious liver injury and inflammation despite their normal ALT levels. Significant fibrosis or moderate hepatocytes injury is markers for beginning antiviral therapy in CHB patients with normal ALT levels.^25^ On the basis of current guidelines,^26^ these patients will not receive antiviral therapy without a liver biopsy to confirm the presence of the suspected liver pathology. However, liver biopsy is an invasive procedure that is accompanied by a nonnegligible risk of complications. Thus, to minimize the need for biopsies, it is important to explore additional markers that can compensate the deficiencies of current ones. In this study, we investigated GP73 in the livers and serum of 1150 patients with chronic HBV infections. Our data suggest that GP73 could be a useful marker for the detection and prediction of liver disease and liver disease severity in patients with chronic HBV infection.

The primary finding of this study is that changes in both GP73 liver expression and serum levels were positively correlated with changes in hepatic necroinflammatory activity in CHB patients. Hepatic GP73 expression and serum 73 levels rose in parallel with hepatitis severity, from nonexistent or mild to severe hepatitis. This finding confirmed recent reports demonstrating detection of GP73 expression in both acute and chronic liver diseases, relating elevated serum GP73 levels to hepatic inflammation and fibrosis.^18,19,27^ Neither the HBV infection nor the replication process seems to increase GP73 expression in the liver or trigger release of more GP73 into the blood. This was demonstrated by our observation that few hepatocytes expressed GP73 and that serum GP73 levels were low in HBV-C without liver injury. However, once hepatic necrosis was triggered, the affected hepatocytes started releasing more GP73 into the blood, resulting in elevated serum GP73 concentrations. In the meantime, an inflammatory reaction, including the influx of infiltrating lymphocytes, occurred at the necrotic sites. The infiltrated immune cells may produce and release higher concentrations of proinflammatory cytokines in the liver. In response to this increased inflammatory environment, or in response to other pathologic changes such as fibrosis and carcinogenesis, hepatocytes may increase the expression of GP73. This process would explain why hepatic GP73 expression and serum GP73 levels were significantly increased in patients with severe hepatitis, cirrhosis, and HCC.^19,20^ In addition to liver injury-mediated GP73 release from hepatocytes, other mechanisms, including induction and regulation by interleukin-6 or interferon-γ, may facilitate GP73 release.^11,28^

A consensus finding from recent studies is that the majority of hepatocytes started expressing GP73 in livers experiencing acute or chronic hepatitis.^17^ In addition to hepatocytes, GP73 mRNA and protein levels were elevated in the activated hepatic stellate cells in patients with chronic liver diseases.^19^ The expressions of both GP73 and endoprotease were significantly upregulated in injured hepatocytes and activated hepatic stellate cells, leading to increased GP73 release into the circulation.^29^ Such results support the positive correlation between elevated GP73 expression in the liver and progressive liver injury. What is lacking is the evidence whether the elevated GP73 expression returns to a normal range along with improving histopathology in the livers, and a study of GP73 expression and serum level kinetics is needed to answer this question. We measured the number of cells expressing GP73 and the expression intensity of those cells before and after the treatment and observed that GP73 expression was significantly reduced by the end of 1 year of ETV therapy. Serum GP73 levels dropped markedly within 3 months of beginning therapy. Our findings were consistent with a report from Iftikhar et al.^19^ Another study^30^ observed that serum GP73 levels were significantly reduced in patients with viral or autoimmune hepatitis in response to antiviral or immune-inhibitory therapy, and these authors suggest using GP73 as a marker to monitor the efficacy of hepatitis treatment. It appears that GP73 expression is activated in hepatocytes in response to microenvironmental changes, such as necroinflammation and fibrosis. If the diseased microenvironment was improved or returned to a healthy state, hepatic GP73 expression and serum levels would also return to a normal range. Our multivariate regression analysis identified the serum GP73 as an only independent factor for predicting hepatic inflammation and fibrosis. Thus, GP73 expression could be used as a prognostic marker for chronic liver diseases.

Nucleos(t)ides (NAs)-based antiviral therapy can block the progress of liver injury and associated complications in patients with chronic hepatitis B. However, not all chronic HBV infection patients are suitable for NAs therapy. The guidelines recommend CHB patients with abnormal ALT or pathological evidence of moderate-to-severe active necroinflammation or a minimum of moderate fibrosis for NAs treatment.^22,26^ However, in some CHB patients, ALT levels may remain within the normal range whereas the liver experiences active necroinflammation or liver fibrosis. Our study showed that 29% and 33% of HBV-C patients demonstrated hepatocyte injury (≥G2) or significant fibrosis (≥S2) in their biopsies, respectively. These patients would not have qualified for antiviral treatment on the basis of their normal ALT scores. Therefore, additional liver injury biomarkers are urgently needed. The data from this study, as well as others, suggest that serum GP73 levels rose in parallel with increasing necroinflammation intensity and fibrotic activity. Furthermore, GP73 was elevated in HBV-C patients who showed normal ALT levels despite hepatocyte injury (≥G2) or significant fibrosis (≥S2). Thus, serum GP73 appears to be a more sensitive biomarker of hepatic injury, inflammatory response, and fibrosis than ALT and can compensate for ALT deficiency in patients with HBV-C. The combination of serum GP73 with other biochemical markers could improve CHB patient management in 2 respects: first, the combination would reduce the possibility of missing the correct diagnosis of antiviral treatment eligible patients, and second, patients with progressive fibrosis would be alerted.

This study showed that serum GP73 in the LC group was significantly higher than in the HCC group. Our results were consistent with some previous reports,^31–33^ but differed from others^14,16,17^ in which higher serum GP73 levels were found in HCC when compared with LC patients. The relative difference in serum GP73 levels between LC and HCC patients may simply reflect slight variations among the different studies concerning the staging of liver diseases. More important is that the serum GP73 level detected was significantly increased in both HCC and LC groups, making the differences between the two groups relatively unimportant. When testing the clinical utility of the serum GP73 detection for HCC and LC diagnosis, the probability of detecting HCC patients was significantly increased when we set the cut-off value of GP73 at 153.60 ng/mL for HCC. If the cut-off value was set at 138.20 ng/mL, GP73 appeared to be an effective marker for diagnosing LC. Thus, GP73 detection in the serum can function as a complementary marker for HCC and LC detection. In combination with other markers and tests such as ALT, AFP, ultrasound, and gastric endoscopy, GP73 detection may help rule out early-stage LC or HCC in chronic HBV-infected patients. The specificity of serum GP73 for HCC diagnosis in our cohort was 68.11%. The 68.11% specificity falls within the range of 60.3% to 75% reported by some studies,^14,34,35^ but it is clearly lower than the specificity reported for other cohorts that exhibited 93.5% to 97.4% specificity.^16,36^ This significant discrepancy in GP73's specificity in HCC diagnosis can be partly explained by differences in the disease stages of patients, but may also be a result of the different antibodies used for the ELISA in each study. In addition, GP73 protein may consist of several subtypes, some of which may show higher HCC detection specificity.^37^ A number of challenges remain before GP73 can be used as a biomarker for HCC, including inconsistent specificity, insufficient sensitivity, and nonsatisfactory value for early diagnosis of HCC.^38,39^ Therefore, further evaluation should be carried out in large multicenter-based cohorts.

In conclusion, changes in GP73 expression in the liver and serum parallel changes in the severity of chronic liver diseases. Hepatic GP73 expression and serum GP73 levels were elevated in HBV-C patients with normal ALT levels despite the presence of necroinflammation and fibrosis. GP73 appears to be a more sensitive biomarker than ALT for liver injury detection. In patients treated with ETV, GP73 serum levels were reduced in response to the mitigation of liver injury. The results of this study indicate that serum GP73 can be used as a prognostic marker for chronic liver diseases.
